# Treatment of Fractures of the Femur

**Published:** 1918-09

**Authors:** Joseph A. Blake


**Treatment of Fractures of the Femur.** By Lieutenant-Colonel Joseph A. Blake, M. C. Colonel Blake said in part :

As Major Sinclair has said, the treatment of fractures of the femur naturally falls under three heads, each of almost equal importance, namely : transport, operative treatment, and mechanical treatment by splints or apparatus.

In regard to transport, the American Army has taken the ground that the wounded should be splinted on the field where picked up, and the splints for this purpose have been designed to occupy as small a space as possible and to be readily carried on to the field. This is the reason for adopting the folding half Thomas splint for fractures of the femur; and the transport of fractures has been so admirably and effectively accomplished under the careful teaching of the officer in charge, that the use of this splint is going to be continued, although it requires more skill to put it in place than does the full ring splint.

Although the splinting on the field has been well done, the same cannot be said of the splinting at the evacuation hospital, and, with due consideration of the stress under which the surgeons were working during the last few weeks, there has been room for much improvement. Every surgeon should make himself proficient in the use of the Thomas splint and it is not putting it too strongly to say that no surgeon should be promoted until he is able to put one on correctly. Until the surgeons at the Field and Evacuation Hospitals become more skilful it might be well to send teams to these hospitals whose only duty should be to see that the personnel are instructed and the splints properly applied.

There should be as little transport as possible for fractures of the femur. Unfortunately they have to be transported and it is important to know at what periods of their treatment transportation is the least harmful. If it were not for delaying the primary operation the best time would be before the operation. The next least harmful period is immediately after the operation before repair of the soft parts has commenced. Transport during the period of consolidation is extremely harmful.

Snug circular bandages should never be used for injuries of the thigh as swelling is more than apt to cause constriction, not infrequently promoting gas gangrene and leading to amputation.

The primary operation differs according to the time when it is performed. When done in the first eight hours before the bacteria have commenced to multiply throughout the wound, it should be confined to a paring away of devitalized tissue. Only detached fragments of bone should be removed, with the exception that in contamination of the medulla in fractures, when the wound is made by penetrating missiles, enough of the fragments should be removed to permit cleansing the medulla, but no more.

Primary suture should only be attempted in the most exceptional instances, and in these only when the patient is to be kept in the service of the operator and not evacuated.

Operations in fractures already infected must be done in a different way. The incisions must be long enough to expose fully the injured muscles and they must be inspected for some distance beyond the original wound. All muscle tissue showing signs of commencing gas gangrene must be excised. The wounds must be left widely open.

While not touching upon the indications for amputation, one must protest against guillotine amputation. It sacrifices an inordinate amount of skin, thus always necessitating a secondary amputation and it is well known that the skin plays no part in the dissemination of gas gangrene. It is believed that as far as drainage is concerned, it is inferior to the transfixion amputation by long anterior and short posterior flaps. In the latter method lateral incisions can be extended upward affording efficient drainage, or the posterior flap may be split without endangering the character of the stump as the bone is covered by the anterior flap alone.

The question of internal fixation is always arising in the operative treatment of gunshot fractures of the femur. In general it is to be deprecated. Occasionally in oblique fractures when there is fear that the fragments may become displaced in the interval between the operation and the adjustment of the limb in the apparatus for definitive treatment, it may be justifiable to use an encircling wire which is to be removed within a few days. Methods which cause additional traumatism, and particularly perforations of the cortex through which infection may reach the medulla, should not be used.

The principles of mechanical treatment have been so admirably expressed by Major SINCLAIR that little can be added. His points in regard to bending the splint and reducing the fracture by using the bands stretched across the splint as a fulcrum are particularly valuable.

For traction I have always preferred the weights and pulley, believing that it is the only way by which the exact amount of traction can be determined; also to permit varying the amounts of traction according to the requirements of the case.

For instance a heavy weight and traction can be applied at the outset to reduce the fracture and then diminished to stress just sufficient to retain position. Used in this way the traction may be so diminished during the latter part of the treatment as not to cause any stretching of the ligament of the knee.

As to the method of applying traction, I use glued bands, Ramshoff's tongs, and the Codavilla pin. Direct traction with the tongs on the femur has been the most satisfactory. It is the most efficacious and does not interfere with massive movements of the knee. The Codavilla pin is used in cases in which the wounds are so low as to prevent the employment of the tongs. In these cases it is placed through the tibia just behind the tubercle. It has the disadvantage of pulling below the knee joint and preventing passive motion of the knee. It possesses no great advantage over the glued bands and is only used when the latter have caused blistering.

For splints the Thomas is the best except in high fractures with wounds about the glutial fold. In these cases the Hodgins is preferable. In using the Thomas it is bent to suit the case, the amount of bending depending not only upon the level of the fracture, but upon the manner in which traction is applied. The axis of traction should be more horizontal than that of the femur over the cross bands of the splint. Therefore when using the tongs or pin, the femur has to be more flexed than when using glued bands and therefore the splint has to be bent so as to produce the desired flexion. We have found that it is best to bend the splint a little above the level of the knee.

Counter balance suspension has been found to be a great help. With it the patient has a greater latitude of motion in bed; and in many cases of hip wounds, by means of a counterbalanced sling under the body, the patient may readily be suspended in the air without disturbing his fracture.

**Treatment of Fractures of the Femur.** By Medecin-Major LERICHE.

